# A large-scale, gamified online assessment of first impressions: The Who Knows project

**DOI:** 10.3758/s13428-025-02601-w

**Published:** 2025-02-03

**Authors:** Richard Rau, Michael P. Grosz, Mitja D. Back

**Affiliations:** 1 Institute for Mind, Brain, and Behavior, HMU Health and Medical University Potsdam, Potsdam, Germany; 2 University of Münster, Münster, Germany; 3 JICE, Joint Institute for Individualisation in a Changing Environment, University of Münster and Bielefeld University, Bielefeld, Germany

**Keywords:** Interpersonal perception, Personality judgment, Accuracy, Gamification, Online assessment

## Abstract

Interpersonal judgments play a central role in human social interactions, influencing decisions ranging from friendships to presidential elections. Despite extensive research on the accuracy of these judgments, an overreliance on broad personality traits and subjective judgments as criteria for accuracy has hindered progress in this area. Further, most individuals involved in past studies (either as judges or targets) came from ad-hoc student samples which hampers generalizability. This paper introduces *Who Knows* (https://whoknows.uni-muenster.de), an innovative smartphone application designed to address these limitations. Who Knows was developed with the aim to create a comprehensive and reliable database for examining first impressions. It utilizes a gamified approach where users judge personality-related characteristics of strangers based on short video introductions. The project incorporates multifaceted criteria to evaluate judgments, going beyond traditional self-other agreement. Additionally, the app draws on a large pool of highly specific and heterogenous items and allows users to judge a diverse array of targets on their smartphones. The app's design prioritizes user engagement through a responsive interface, feedback mechanisms, and gamification elements, enhancing their motivation to provide judgments. The Who Knows project is ongoing and promises to shed new light on interpersonal perception by offering a vast dataset with diverse items and a large number of participants (as of fall 2024, *N* = 9,671 users). Researchers are encouraged to access this resource for a wide range of empirical inquiries and to contribute to the project by submitting items or software features to be included in future versions of the app.

## Introduction

Interpersonal judgments are pervasive and paramount in the intricate landscape of human social interactions. From the moment individuals encounter one another, their minds instinctively engage in a continuous process of assessing and evaluating one another's traits, goals, and behaviors. As pointed out by Funder and West, consequential decisions such as “(…) whom to befriend, trust, avoid, hire, promote, release from prison, or elect as president” result from this process (1993, p. 458).

Although past research has yielded many valuable insights into the (in)accuracy of interpersonal judgment, the methods used to assess these judgments have had weaknesses. Here, we introduce an ambitious and innovative endeavor, the Who Knows project, that seeks to remedy some of these weaknesses and create a large and reliable database for (re)examining a variety of research questions surrounding first impressions. The core element of the project is a gamified smartphone application in which users try to infer personality-related information of strangers based on short video introductions. In the following, we briefly summarize the project’s theoretical background and then provide a detailed overview of how the Who Knows app is designed, what type of samples and data are collected, and what kind of research questions can be answered with these data.

## Background

The topic of interpersonal perception, impression formation, or personality judgment has a long and complex tradition in cognitive, social, and personality psychology. Different lines of work have focused on different aspects of the topic such as the *errors* surrounding personality judgments vs. the *processes* behind these judgments vs. the *accuracy* of these judgments. And even among scholars with a focus on accuracy, a variety of methodological and philosophical debates have kept different approaches to studying the same topic isolated from one another (for an historic overview, see Funder, 1995). The current project takes a *realistic* approach to accuracy, which comes with two fundamental assumptions. The first assumption is that when determining whether a given judgment of a target person’s trait is accurate, there does in principle exist an *objective truth*. The second assumption is that *multifaceted criteria* are needed to approximate this truth (Funder, 1995). One may determine the accuracy of an interpersonal judgment by means of self-other agreement, other-other consensus, or successful behavioral prediction, whereas “(…) no one can hope to gather all the criteria one might desire in any particular study. But the goal (…) is always to gather as many criteria as you can and for the literature as a whole not to restrict oneself to just one or a few criteria for accuracy.” (Funder, 1995, p. 657).

Much work from the past decades has studied the accuracy of person judgments by means of self-other agreement (Krzyzaniak & Letzring, 2021). Thus, these studies accept (at least implicitly) that there is an objective truth underlying personality traits and they assume that targets’ self-reports about these traits are a valid criterion for this truth. Even though this claim is certainly defensible, it seems problematic in the field of accuracy research where impressions are typically assessed on (ultra-)short personality inventories to capture broad personality domains such as the Big Five (John & Srivastava, 1999). These inventories have the benefit of efficiently covering a relatively wide scope of personality content, but the abstractness of the items in these inventories opens up unwanted room for interpretation. Further, many abstract personality items are also rather evaluative which introduces potential bias due to socially desirable responding (Paulhus, 2002). Together, this may render self-other agreement a non-optimal criterion for accuracy. The Who Knows app does not draw on a standardized inventory but instead assesses judgments regarding a large variety of personality content, often by means of tasking perceivers with predicting tangible, specific aspects of the targets’ everyday lives. This is done using both conventional Likert-type ratings and forced-choice formats. As such, our approach complements the common approach that focuses exclusively on broad personality ratings.

A related issue in most of the existing literature on personality judgment is that the field adheres only insufficiently to the call for multifaceted criteria (Funder, 1995). Specifically, behavioral prediction is rarely ever used as an accuracy criterion due to feasibility constraints (Krzyzaniak & Letzring, 2021), constraints that also exist in the present research. Nevertheless, by including *knowledge* items in addition to typical self-concept items, the present research also features an accuracy criterion that taps the targets’ actual performance (i.e., whether they correctly answered specific trivia questions during their interview) as opposed to their self-reported beliefs, desires, or competencies. As such, the current project complements existing approaches to accuracy by including predictions of trivia performance as instances of behavioral prediction. To the degree that self-reports are biased (e.g., due to socially desirable responding), the behavioral prediction approach offers a more valid measure of accuracy.

Another shortcoming of many existing studies on accuracy concerns the sampling of participants. The common study design is a so-called *Round Robin* where participants are part of a group, usually comprising between 4 and 10 individuals, in which everyone rates everyone else (Kenny, 1994), usually after having interacted face-to-face with them in some sort of icebreaking game or problem solving task (e.g., Anderson & Kilduff, 2009; Rau et al., 2022a; Srivastava et al., 2010) or in a series of dyadic “speed-dates” (e.g., Carlson, 2016; Elsaadawy et al., 2021; Human et al., 2019). This design has the great advantage of being statistically efficient (since every participant acts both as a perceiver and as a target) and highly ecologically valid (since judgments are based on actual, personal interactions).

However, one downside of the Round Robin design is that the necessity to recruit participants in groups implies that the reliability of individual differences in accuracy will be limited by the size of the groups (Bonito & Kenny, 2010). Furthermore, the generalizability of findings will often be limited because most studied Round Robin groups are more homogeneous in terms of age, education, or occupation than a group of randomly drawn people from the population of interest would be. In fact, given that recruiting truly heterogeneous face-to-face groups is hardly feasible in most research settings, there seem to exist essentially no Round Robin studies in the literature that investigate how people judge people who come from a different socio-demographic stratum than themselves. The Who Knows project addresses this shortcoming by avoiding a Round Robin design and instead allowing people to participate as targets (i.e., by showing up to an in-person video interview) separately from the possibility to participate as perceivers (i.e., by downloading and using the Who Knows app). This offers greater control over the sampling process compared to traditional Round Robin research. Once a sample of targets that meets one’s criteria for sociodemographic diversity has been implemented to appear in the app, the problem of measuring accuracy for an overly homogenous group of people is essentially solved. Regarding the sample of perceivers, sociodemographic diversity can be pursued by how and where the Who Knows app is advertised. In general, its decentral nature as an online platform allows for a crowdsourcing-style data collection and is expected to yield a much larger and cost-efficient database than could ever be collected in any on-site study.

## Open questions

Although personality judgment has received a lot of attention from empirical researchers during the past decades, answers to several basic questions have remained inconclusive. For example, a recent review identified (among others) the following open questions (Letzring et al., 2021): First, what *substantive* target characteristics underly people’s *judgments* of these characteristics? Second, what characterizes individuals who make particularly accurate judgments and is this ability a consistent individual difference across judgment domains (cf. the *good judge hypothesis*; Funder 1995)? Third, can the ability to make accurate judgments be *trained*? One reason why these questions have remained insufficiently answered may be an overreliance on short personality inventories capturing a few broad content domains. With its very large pool of highly concrete and heterogenous items, the present work aims to increase the likelihood of judgements being driven by substantive considerations rather than interpretative or evaluative biases. Furthermore, because the used items are domain-specific but the pool of items cover a broad range of domains, the Who Knows project allows for a domain-specific as well as a domain-general investigation of between-judge differences in accuracy. Finally, through its high degree of gamification, the Who Knows app leverages users’ intrinsic motivation and produces long time series data (e.g., several hundred judgments provided by the same user) and thereby enables an investigation of training effects, a possibility that seems unrealistic to achieve in conventional study designs because of excessive response burden.

Answering these open questions will not only advance our theoretical understanding of person judgments but also have implications for applied settings. For example, social psychologists have long known that first impression are heavily influenced by stereotypes, that is, by assumptions about what traits are shared by most exemplars of some social category. However, much less is known about a potential “kernel of truth” (i.e., substance) in stereotypes and it remains to be seen how harmful (or helpful) in terms of accuracy it is for judges to rely on them when forming impressions of particular, actual target persons. Concerning the “good judge hypothesis”, if people were found to consistently differ in how good of a personality judge they are this would have important implications for hiring practices in areas such as health care, jurisdiction, or education because the impressions formed by professionals in these fields have particularly far-reaching consequences. Concerning training effects, the same professionals would make for a highly relevant audience of potential intervention programs if accuracy were found to be trainable.

## The Who Knows App

**Fig. 1:**
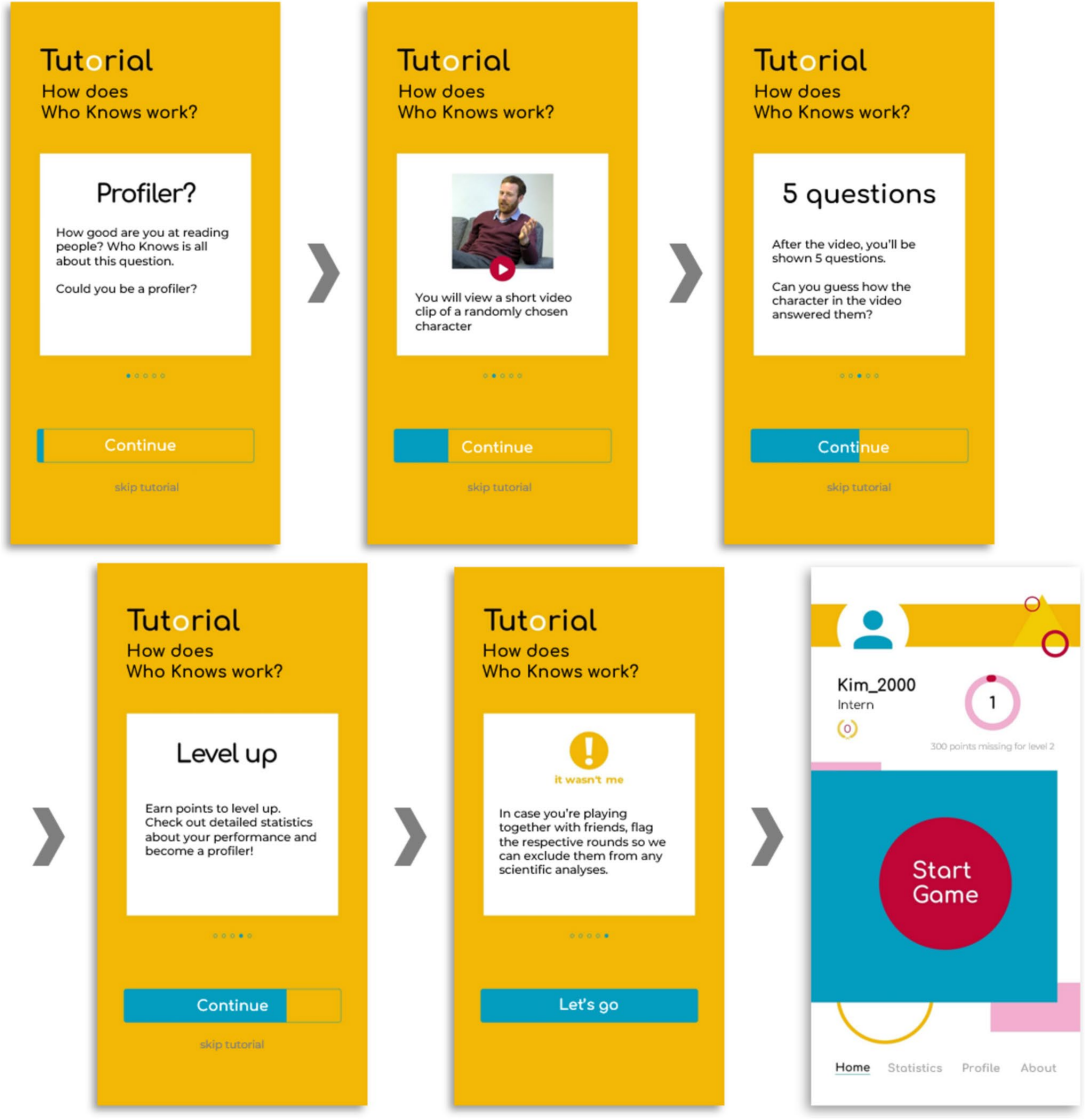
Tutorial and Start Screen of the Who Knows App. *Note.* Upon having set up an account, these screens are the first to be viewed by a novel user. All texts are free translations from the German original

**Fig. 2:**
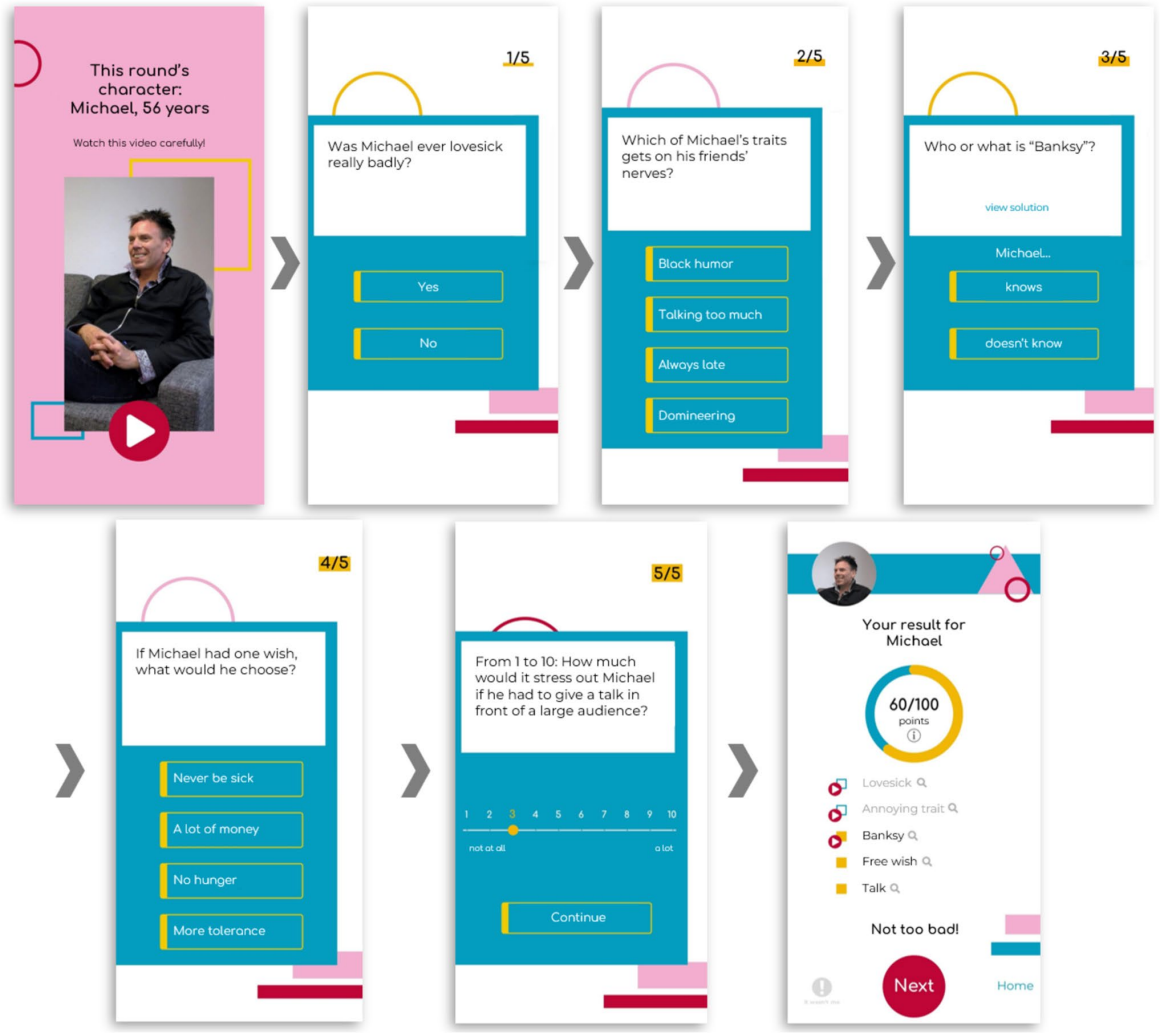
Exemplary Game Screens of the Who Knows App. *Note.* Hitting the play button in the top left screen starts the target’s introduction video. The following five screens record the user’s judgments regarding random items that have been answered by the target. The bottom right screen displays the user’s results for that round. All texts are free translations from the German original

The core data collection tool of the Who Knows project is the Who Knows app. The Who Knows app incorporates a smartphone-based online assessment of realistic first impressions via a quiz application that is easy and fun to use. Users who are interested in learning about their ability to form accurate first impressions of strangers can register for free and get to know real target persons in short video clips. Users are then asked to answer five random questions about the target and then learn how the target has responded to these questions themselves. Figure 1 displays how the game is explained to the users on the tutorial screens within the Who Knows app. Figure 2 shows the screens of an example round about the target “Michael”. Interested readers are referred to https://whoknows.uni-muenster.de to actively try out the app themselves.

The primary goal during the development of the app was an optimal user experience that maintains a high level of engagement and thereby maximizes the users’ intrinsic motivation to provide a large number of judgments. To this end, the app uses a modern and colorful layout and a responsive interface including, for example, micro-animations when the user hits buttons and sound effects when the user earns points.

As shown in the bottom right screen of Fig. 2, the user receives detailed feedback after each round which includes information on the (in)correctness of their judgments (see empty boxes for the items “Lovesick” and “Annoying trait” vs. yellow boxes for the items “Banksy”, “Free wish”, and “Talk”) as well as an opportunity to explore the target’s responses more deeply, either by hitting the magnifier icons (which display the target’s responses in written form) or by hitting the red play-buttons (which play videos of the target answering the questions – an option that exists for roughly two thirds of the items). Further, users can receive feedback on their general performance after having played a certain number of games. This includes feedback on their overall hit rate which is contextualized by also providing the hit rates of the average user and the top ten users. Further, users can receive detailed feedback on their hit rates for specific target groups (male vs. female targets; younger vs. older targets) and for specific content domains (e.g., values and attitudes vs. knowledge and skills). Finally, they can browse their personal game history and re-inspect their and the targets’ responses from rounds played in the past.

Another measure to keep users engaged is a high degree of gamification. Users not only earn points for each correct response they give, they also collect “medals” for each perfect round they play (i.e., for answering all five questions about a target correctly) and they are assigned experience levels. Some of the feedback mentioned above can only be accessed by users with a certain experience level. Further, at certain points, users are required to complete surveys assessing sociodemographic and personality information about them in order to continue playing or unlock access to further features of the app. Table 1 provides an overview of the requirements users need to meet in order to receive access to all of the app’s features.

**Table 1:** Features and Requirements of the Who Knows App

Feature	Requirement within the app
Play up to 10 games	Setup free account
Play up to 30 games	Complete first survey
Play 700 + games	Complete second survey
Get access to feedback on personal overall hit rate	Reach experience level 3 (“Junior Profiler”, usually reached after approximately 13 games)
Get access to detailed feedback (personal hit rates for different subgroups of targets and for different content domains)	Reach experience level 4 (“Senior Profiler”, usually reached after approximately 25 games)
Get access to a browsable history of all games played so far	Experience level 5 (“Mindreader”, usually reached after approximately 42 games)
Option to personally select specific targets to appear in the next game	Experience level 6 (“Guru”, usually reached after approximately 66 games)

## Data

The Who Knows app collects a variety of both behavioral and self-report data. We classify the data into the categories of response data (i.e., the responses users actively provide during games), logging data (i.e., passive data on app usage automatically recorded by the app), and survey data (i.e., users’ answers on questionnaire items).

### Response data

Response data pertain to how a particular user has responded on a particular item about a particular target. They are stored as text strings (e.g., “Yes”, “Always late”, “doesn’t know”, “Never be sick”, and “3” in the example from Fig. 1) and are automatically classified in a corresponding dummy variable called “is_correct” (with 0 indicating a mismatch with the target’s response and 1 indicating a match; e.g., as “0”, “0”, “1,”, “1”, “1” for the example from Fig. 1). Note that on items with a response scale from 1 to 10, responses within an interval of ± 1 within the target’s response are classified as correct. This is done for the purpose of providing transparent feedback within the app. For scientific use, the raw responses may instead be used and be treated as a continuous variable in correlational analyses.

### Logging data

Logging data essentially consist of digital timestamps recording the precise date and time when a user has started to visit a particular screen of the app and when they have left that screen. After a modest degree of data processing, these logging data may speak to various psychological processes taking place during app usage. Table 2 provides an overview of potential use cases for these data.

**Table 2:** Logging Data Collected by the Who Knows App

Logged screen / activity	Explanation	Suggested psychological interpretation
Video player	Time spent watching the target’s introduction video; varies because users are free to pause, replay, or skip parts of the video	Greater time indicates greater attention paid to video content
Item screen	Passed time between item display and click on response button	Greater time indicates more deliberate (vs. intuitive) decision making
View solution to knowledge question	Whether or not user clicked to view the correct solution in case the item was a knowledge question (cf. top right panel in Fig. 2)	Click suggests that the user themselves did not know or was uncertain about the solution
*Score* screen	Time spent on *score* screen of a given game	Indicates the user’s motivation to learn from the feedback provided to them aggregated across all five items of the game
Click on magnifier icon on *score* screen	Whether or not user chose to view the target’s actual answer to the respective question	In case of items with two response options: Indicates that the user cannot remember their response. This may reflect a lack of focus. In case of items with four or ten response options: Indicates either that the user cannot remember their guess or that they are motivated to learn more about the target
Click on play button on *score* screen	Whether or not user watched a video of the target answering the respective question	Indicates a motivation to learn more about the target. A preference to view answers to items where the user was right (vs. wrong) might reflect a motivation to receive self-serving (vs. self-critical) feedback
*About* screen	Time spent on a static page listing references, contact options, etc	Indicates the user’s interest in the scientific background of the app
*Profile* screen	Time spent on a page listing details about one’s account and offering the option to change one’s user name and to sign out	–
S*tatistics* screens	Time spent inspecting feedback about one’s personal overall hit rate (*statistics 1* screen), one’s personal hit rates for different subgroups of targets (*statistics 2* screen) and one’s personal hit rates for different content domains (*statistics 3* screen)	Indicates how strongly a user is motivated to gain insight into their ability to make accurate judgments about others
*Chronic* screen	Time spent browsing one’s game history	–
Re-view score screen of earlier games	Time spent on score screen of a given game	Indicates the user’s motivation to recall or memorize information about the respective target

### Survey data

Survey data are collected at various points during a user’s journey through the app. For instance, after their fifth game, users are asked to provide basic sociodemographic information. Until having played their tenth game, they can opt not to do so but after that, they cannot play any more rounds without providing these data. As shown in Table 3, there are several further (mini) surveys covering a variety of psychological constructs which have to be completed before users reach unlimited access to the app. Some of these assessments are implemented into the Who Knows app and others have been outsourced to an external surveying platform (i.e., formr, Arslan et al., 2020). Since not all users make it this far and since some of the items were moved to a different survey or added after the app had been launched, the number of users who have completed an assessment varies from construct to construct (see rightmost column in Table 3). A spreadsheet including the original wording of items and response options presented in the external surveys can be retrieved from https://osf.io/rdf7u/.

**Table 3:** Survey Data Collected in the Who Knows Project

Source	Construct (response format)	Number of items	Number of respondents (as per fall 2024)
In-app assessment (completion mandatory after game #10)	Age (continuous)	1	5106
	Gender (3 categories, single choice)	1	5106
	Education (5 categories, single choice)	1	4369
	Living situation (4 categories, single choice)	1	4660
External survey #1 (completion mandatory after game #10)	Political orientation from left to right (1–9)	1	4114
	Religiosity (1–6)	1	4109
	Mental health problems in past year (yes / no / prefer not to answer)	1	4093
	Mental disorders (checklist of 12 items; only presented if mental health problems = yes)	1	1399
	Hours spent on smartphone on typical day (0–5)	1	4102
	Grade point average (1–5)	1	3289
	Psychology student (yes / no; if education = = A levels)	1	1005
	Semester (1–20; if psychology student = yes)	1	182
	Has psychology degree (yes / no; if education = high school degree)	1	2284
	Self-reported IQ (only assessed from participants who indicated they had completed a professional IQ test in the past; 40—160)	1	579
	Occupational status (10 categories, single choice)	1	3907
	Income (5 categories, single choice)	1	3481
	Geographic region of primary socialization (5 categories, single choice)	1	2798
	Motive for using the app (5 categories, multiple choice)	1	3901
In-app assessment (required before receiving feedback on personal hit rate for the first time)	Self-perceived quiz performance relative to the average user (1–5)	1	1561
External survey #2 (completion mandatory after game #30)	Self-esteem (1–6; Brailovskaia & Margraf, 2020)	1	1744
	Loneliness (1–6)	1	1731
	Emotional validation during childhood (1–6)	1	1731
	Satisfaction with life (1–6; Lucas & Donnellan, 2012)	1	1743
	Narcissistic admiration / rivalry (1–6; Leckelt et al., 2018)	3 per subscale (α = 0.73 / 0.61)	1718
	Interest orientation (6 separate items concerning realistic, investigative, artistic, social, enterprising, and conventional orientation rated from 1–6; Holland & Rayman, 2013)	1 per subscale	1723
	Values (10 separate items concerning power, achievement, hedonism, stimulation, self-direction, universalism, benevolence, tradition, conformity, and security rated from 1–6; Lindeman & Verkasalo, 2005)	1 per subscale	1723
	Certainty about mental states of self / others (1–7; Müller et al., 2023)	3 per subscale (α = 0.76 / 0.70)	1718
	Big Five: Extraversion, agreeableness, conscientiousness, neuroticism, openness (1–5; Rammstedt et al., 2020)	6 per subscale (α = 0.75 / 0.69 / 0.76 / 0.81 / 0.69)	1684
	Self-insight motive (1–6)	5 (α = 0.86)	1537
	Level of personality functioning (1–5; Spitzer et al., 2021)	4 (α = 0.74)	767

### Measures to secure high data quality

The rationale behind gamifying the Who Knows app is to collect data from participants who are intrinsically motivated. They use the app not because they are paid for doing so but because they enjoy learning something about themselves and others and perhaps because of an ambition to increase their score or level. As they lose interest, get tired, etc., they can simply stop using the app. For these reasons, one might expect the collected data to be of high quality. In contrast, a possible concern may be that framing the tool as a game might undermine participants’ seriousness. For instance, participants might use the app in groups (i.e., decide on their responses jointly with friends or family) or might even hand their device to another person and have that person respond instead of themselves. Also, participants may provide invalid reports on sociodemographic questions in case of privacy concerns or they might engage in careless responding in the surveys in case they want to unlock the app to play more game rounds as quickly as possible.

To learn whether these concerns are warranted, we implemented several data quality checks within the Who Knows app and at the end of the external surveys. Specifically, users are encouraged to flag any game rounds which they have not completed alone (see “it wasn’t me” button in Figs. 1 and 2) such that these rounds can be excluded in any data analyses. Second, users can indicate that their responses to sociodemographic questions should not be used for scientific analyses. This allows for an exclusion of data which are potentially invalid because of participants’ privacy concerns. Finally, verification questions occur at the end of the two external surveys such that users must indicate whether they responded seriously vs. recommend not to use their data. Overall, the results of these quality checks suggest that instances of invalid or careless responses are quite rare: Since the implementation of these quality checks, only a small fraction of game rounds have been flagged by users hitting the “it wasn’t me” button (0.8%) and the majority of in-app responses to sociodemographic questions (91.1%) as well as external survey responses (survey 1: 93,7%; survey 2: 96,3%) have been declared serious and usable for scientific purposes. In sum, the quality of data collected in the Who Knows project appears to be generally high and is enhanced still by the possibility to exclude data points for which users have indicated a lack of seriousness in their responses.

### Samples

In the Who Knows project, sampling occurs on three independent levels. First, there is a sample of app users who provide judgements of the targets that appear in the app. Since the project is ongoing, the sample of users grows continuously. Second, there is a sample of targets. This sample was actively stratified during the development of the app in order to warrant a certain degree of representativeness. Finally, there is a sample of items. This sample was deliberately generated such that it covers a wide array of personality content. When analyzing Who Knows data, we recommend modelling users, targets, and items as random factors to maximize the robustness and generalizability of statistical inferences (Judd et al., 2017; Yarkoni, 2020). Below, each of these samples is described in detail.

### Users

**Fig. 3:**
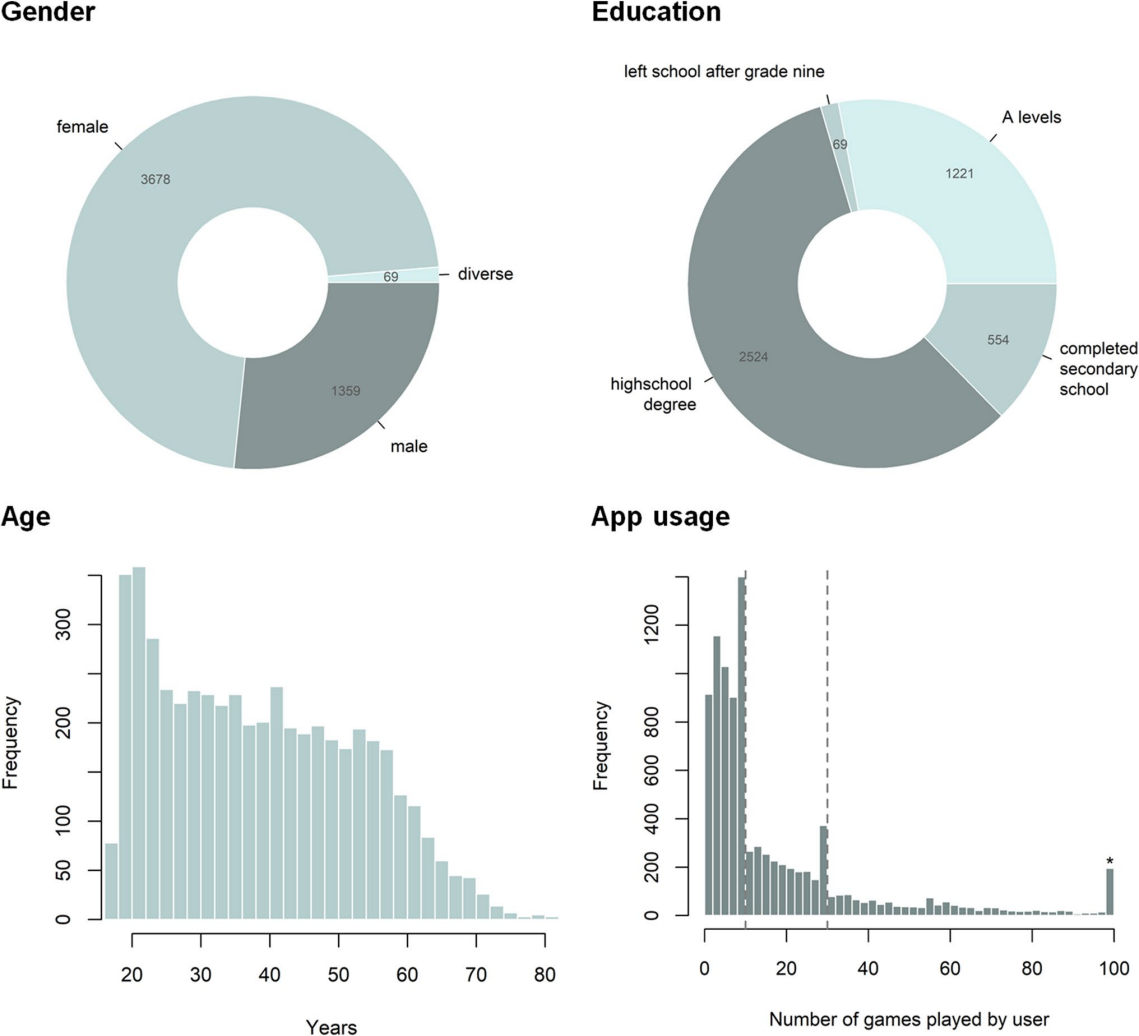
Descriptive Statistics of Who Knows Users as of Fall 2024. *Note.* Dashed lines in the bottom right plot indicate where users are required to complete surveys before being able to continue playing. *open-ended category, includes users who played up to 791 games

As of fall 2023, *N* = 9,671 users have set up an account (excluding test-users) for the Who Knows app and *n* = 5,135 have completed the first survey assessing basic sociodemographic self-descriptions. Their sociodemographic characteristics and app usage frequencies are displayed in Fig. 3. They were recruited via mailing lists, social media posts, and occurrences in public media such as podcasts and magazines. During their account setup, users are informed that their participation is voluntary and that their anonymous data will be used for scientific purposes.

As in most convenience samples in psychology, the majority of the user sample is female and highly educated. Somewhat other than in most psychological research, the sample covers a wide age range that also includes many participants of middle and high age. As such, the sample of users is imbalanced in the sense that some sociodemographic groups are overrepresented but it nevertheless is heterogenous in the sense that individuals from underrepresented groups still do exist in the sample. This makes it possible to use techniques like poststratification (Holt & Smith, 1979) or inverse probability weighting (Seaman & White, 2013) to derive unbiased estimates of population parameters.

On average, users played *M* = 19.6 game rounds (*SD* = 31.9) but as can be seen in the bottom right panel of Fig. 3, the distribution is highly skewed such that the majority of participants played just a few rounds whereas a minority of users played up to several hundred rounds. The plot also shows that some users stopped using the app at points where they were required to complete one of the external surveys (i.e., after games #10 and #30). Although the number of users as well as the number of rounds played by these users are still growing, the data collected so far should already provide excellent statistical power for most research questions concerning between-user differences such as antecedents and consequences of perceptive accuracy. As an example, if one were to investigate whether age differences may explain who is good and who is poor at judging people accurately, the current data would provide statistical power of 99% to detect such an effect even if it was as small as *r* = 0.06. All in all, the app has recorded over 700,000 judgments as of fall 2024.

### Targets

**Fig. 4:**
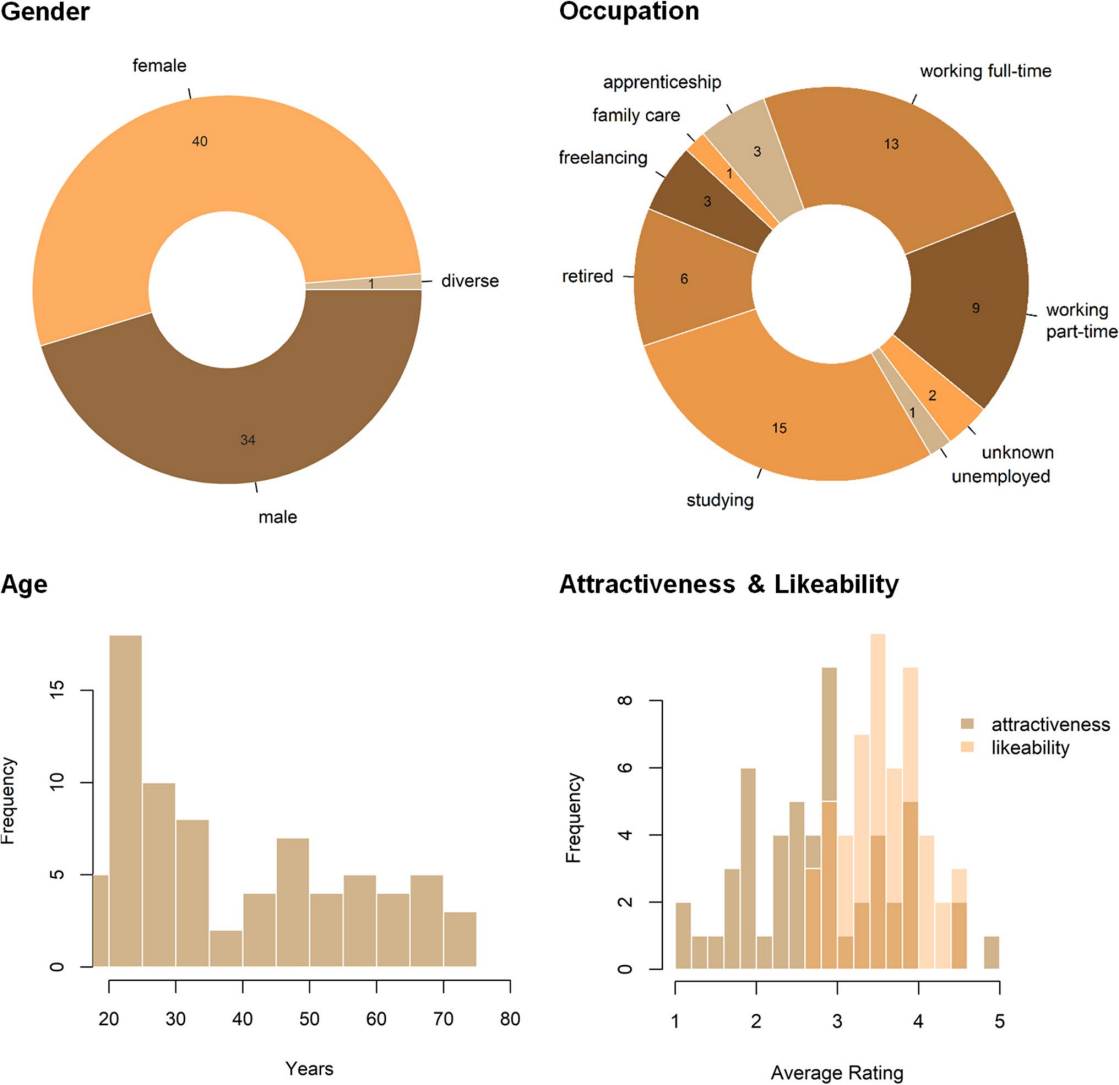
Descriptive Statistics of Individuals Appearing as Targets in the Who Knows App

As of fall 2024, the app features 75 targets. They were recruited and interviewed in the German city of Münster and provided informed consent for using their first name, age (at the point of the interview) and video material in a public web-application. A first wave of recruitment took place in 2021 (*n* = 53) and a second wave in 2023 (*n* = 22). They were recruited via flyers, wallpapers, mailing lists, and local newspaper announcements. Targets were compensated with 20€ and received item-level feedback on the impressions formed about them by the app users. The first 50 participants also took part in a lottery of 1*500€. To obtain a sample of targets as diverse as possible in terms of gender and age, we loosely stratified the sample. Specifically, we required the sample to be approximately evenly distributed across male and female gender and across groups of younger age (below 30 years) and older age (at least 30 years). Figure 4 displays the sociodemographic characteristics of the target sample.

During the video-recorded interview, targets first provided a brief, semi-structured self-introduction in which they stated their name, profession, hobbies, interests, relationship status, as well as three adjectives that described them the best. They were then asked a random sample of 100 questions from the pool of 811 items described below. They could respond at whatever level of detail they liked and skip as many items as they liked. After having completed the interview, they could also request for parts of their interview to be deleted from the video. Finally, they completed a questionnaire on sociodemographic information and personality (German Big Five Inventory 2; Rammstedt et al., 2020).

Based on the raw video material, research assistants generated the content meant to appear in the Who Knows app. Specifically, they coded all of the targets’ responses and cut videos of approximately 30 s length that showed a compacted self-introduction of each target. At this stage, they discarded items to which they deemed the target’s responses uninformative (e.g., a target replying “I don’t remember” to the question what was their dream job as a kid). Whenever a response comprised more than just a short statement (e.g., “I always wanted to become a paleontologist because Jurassic Park was my favorite movie for the most part of my childhood” instead of just “paleontologist”), they also saved a video showing that response. For the 53 targets recruited in the first wave, seven trained research assistants (4 female, 3 male) rated the targets’ physical attractiveness and likeability on a scale from 1 (not attractive / likeable at all) to 5 (very attractive / likeable). As shown in the bottom right panel of Fig. 4, physical attractiveness ratings covered a quite wide range whereas likeability exclusively spanned moderate to high values. Inter-rater agreement was excellent for attractiveness ratings, ICC (3,7) = 0.93, and acceptable for likeability ratings, ICC (3,7) = 0.71. The same group of observers also rated the number of times each target mentioned thoughts, feelings, and behaviors, ICCs (3,7) = 0.79, 0.71, and 0.88 respectively, as well as the perceived quality of revealed information (aggregated across six items, α = 0.92), with good inter-rater agreement (average ICC [3,7] = 0.82). In addition, we have recorded the length of these targets’ videos in seconds as well as the number of words spoken on the videos as cues for the quantity of information. All these target-level data can be retrieved from https://osf.io/rdf7u/.

Currently, the target sample size should yield modest to insufficient statistical power for most research questions concerning between-target differences such as antecedents and consequences of expressive accuracy. As an example, if one were to investigate whether age differences may explain who is easy and who is hard to judge, the current data would allow the detection of such an effect with satisfactory statistical power of 80% only if this effect was as large as *r* = 0.33. To address this, we plan to extend the app's capabilities, allowing users to become targets by uploading videos of themselves, rather than requiring an in-person interview at our laboratory. That is, we will develop an interface with which users can upload video content if they wish to become a target. A content moderation system will warrant sufficient audio and video quality and that the content is in accordance with the terms of use. Even if only a fraction of users should opt to become a target that way, this should considerably increase the target sample size and contribute to much greater statistical power for between-target analyses in the future.

### Items

The games within the Who Knows app draw on a pool of 811 highly heterogenous items^1^ covering a variety of response formats and content domains. Example items are displayed in Table 4. In contrast to item sampling strategies common in the context of scale construction, our item pool was generated without any particular factor model in mind. Instead, our primary criterion for generating items was to cover anything that is potentially interesting or entertaining about a target person. As such, a team of research assistants brainstormed for potential items seeking inspiration not only from comprehensive personality inventories but also from TV shows and board games on interpersonal perception. Items were then checked for redundancy, ambiguities, spelling mistakes, etc. and corrected or removed accordingly. All remaining items were kept (i.e., none were excluded based on psychometric properties). The example items in Table 4 are somewhat representative of the entire item pool. A complete list of items can be retrieved from https://osf.io/rdf7u/.

**Table 4:** Example Items from the Who Knows Item Pool

Type	Example Item [*response format*]		Content category
	As read to the target	As displayed to the user	
Binary	Have you ever participated in a political demonstration? [*yes vs. no*]	Has X ever participated in a political demonstration? [*yes vs. no*]	Values and attitudes
	Do you out of bed immediately when his alarm rings? [*yes vs. no*]	Does X get out of bed immediately when his alarm rings? [*yes vs. no*]	Lifestyle and leisure
	Do you write postcards when he is on holiday? [*yes vs. no*]	Does X write postcards when he is on holiday? [*yes vs. no*]	Lifestyle and leisure
Open-ended	What was your first job? [*open-ended response*]	What was X’s first job? [*insurance accountant vs. bartender vs. postman vs. author for children’s books*]	Knowledge and skills
	Which of your traits gets on his friends’ nerves? [*open-ended response*]	Which of X’s traits gets on their friends’ nerves? [b*lack humor vs. talking too much vs. always late vs. domineering*]	Emotions and relationships
	To what kind of charity would you prefer to donate? [*open-ended response*]	To what kind of charity would X prefer to donate? [*historic preservation vs. animal welfare vs. human rights protection vs. environmental protection*]	Values and attitudes
Scale	How much do you like children? [*1* = *not at all, 10* = *very much*]	How much does X like children? [*1* = *not at all, 10* = *very much*]	Emotions and relationships
	How likely are you to overreact in a fight? [*1* = *very unlikely, 10* = *very likely*]	How likely is it for X to overreact in a fight? [*1* = *very unlikely, 10* = *very likely*]	Emotions and relationships
	How important is a healthy diet to you? [*1* = *not important at all, 10* = *very important*]	How important is a healthy diet to X? [*1* = *not important at all, 10* = *very important*]	Lifestyle and leisure
Trivia	What is the capital of Estonia? [*open-ended response*]	What is the capital of Estonia? [*X knows vs. X doesn’t know*]	Knowledge and skills
	What is the difference between Yoga and Pilates? [*open-ended response*]	What is the difference between Yoga and Pilates? [*X knows vs. X doesn’t know*]	Knowledge and skills
	What is 120% of 45? [*open-ended response*]	What is 120% of 45? [*X knows vs. X doesn’t know*]	Knowledge and skills

As can be seen in Table 4, there are four different types of items with distinct response formats. First, 213 items consist of a closed question and are answered in a binary yes-vs-no format both by the target and the user. Second, 154 items consist of questions which were presented to targets in an open-ended format and which are answered by users on a single-choice response format with four options. Among these four options, one reflects the target’s actual answer and the other three are distractors. These distractors are target-specific, that is, they were generated by a team of research assistants who aimed at including at least one distractor that seemed implausible and one that seemed plausible based on consensual, stereotypical knowledge.^2^ Third, 137 items ask for a rating on a scale from 1 to 10, where anchor labels may vary from item to item. Fourth, 307 items concern trivia questions. As described above and other than for the remaining item types, trivia items use the target’s actual performance as an accuracy criterion instead of their self-report. For instance, a user’s prediction that Michael knew what is the capital of Estonia is classified as correct if Michael accurately responded “Tallinn” (rather than “I know”).

For all items but the trivia-type items, an important goal during item generation was that the targets’ self-report could serve as a tangible accuracy criterion. To that end, we tried to avoid references to overly abstract or evaluative concepts but instead tried to come up with items that tapped concrete behaviors or factual aspects of everyday life. As an illustration of this rationale, compare a scenario where a user’s task is to judge whether or not a target described themself as *active* with a scenario where the task is to predict whether the target says they get out of bed immediately when their alarm rings in the morning. In order to master the first task, the user would not only need to assess what they believe to be the target’s actual level of activeness but also consider the target’s potentially idiosyncratic threshold for calling themself *active* (given that activeness is an abstract concept) and the target’s potential tendency to describe themself in a socially desirable fashion (given that activeness is an evaluative concept). In other words, there is the danger of a discrepancy between judging what is objectively true vs. what is the target’s self-report and we assumed that this discrepancy is reduced for items with great specificity and low evaluativeness such as the item in the second scenario.

In addition to abstractness and evaluativeness, we also considered that an item’s response format might influence the validity of self-reports as an accuracy criterion. Whereas classical test theory implies that a reduction of response options translates into a loss of information and, hence, validity (e.g., Gulliksen, 2013; McDonnald, 1999), it has recently been speculated that picking options on multi-point scales may actually open up unwanted space for interpretation and thereby attenuate validity (Roberts, 2018). Empirically, a binary response format in personality items has in fact been shown to produce equally valid measurements compared to traditional multi-point formats (e.g., Simms et al., 2019) which raises the question whether fewer response options may offer a viable, more economical alternative to established Likert-type scales. To obtain the opportunity to test if and when this might be the case in the context of first impressions, we included items of varying response formats.

Finally, as displayed in the rightmost column of Table 4, all items are classified in terms of four broad content categories. The main purpose of this categorization is to provide content-specific feedback concerning a user’s perceptive accuracy in the feedback area of the app. The categorization was done in a top-down manner and was not data-driven.

Even though the current item pool does not adhere to any particular factor model, we are optimistic it is large and heterogenous enough to warrant a decent degree of generalization across the personality space and offers many opportunities for scoring existing personality constructs or constructing new scales based on the collected data. Overall, by including very heterogenous items which often relate to specific behaviors and knowledge, the impressions assessed via the present item pool pertain less to abstract and subjective aspects of self-concept but more to concrete and objective manifestations of personality.

A spreadsheet listing all items can be retrieved from https://osf.io/rdf7u/. Included in this spreadsheet is also the number of times the item has been responded to as of fall 2023 and the average rating of five research assistants concerning the items’ evaluativeness (rated from 0 = *neutral* to 1 = *very evaluative*; *ICC[3,5]* = 0.83; *M [SD]* = 0.26 [0.19]). Note that these ratings were not collected for Trivia items. Further, for all items of type Binary and Trivia the spreadsheet lists the average rating of eight lay persons (four males, four females; *M [SD]*_age_ = 44 [18] years) concerning how a typical male or female would respond to the item. Specifically, they completed two separate assessments (one for each gender) whether a typical male or female would endorse (= 1) or would not endorse (= 0) the item or whether they were undecided (= 0.5). Inter-rater agreement for these ratings was excellent (*ICCs [3, 8]* = 0.94 both for male and female stereotypicality).

## Validity checks

Although it is not the primary focus of this paper to investigate substantive questions, this section will present evidence for the basic validity of the data gathered in the Who Knows project by replicating three central insights from interpersonal perception research based on the data which had been collected until fall 2023. One of each of these insights concerns the level of users, targets, and items.

At the level of users, a common finding in work on “perceiver effects” (i.e., how a perceiver judges the average target; Kenny, 1994) is that perceivers with a more prosocial, communal orientation judge the personality of strangers more positively, that is, more in the direction of the items’ socially desirable poles (Rau et al., 2021, 2022a, b). To replicate this effect, we used 32 items with comparatively high evaluativeness values (16 Binary items, 16 Scale items) to score how much each user tended towards making evaluatively positive vs. negative judgments. Sample items include “Has X ever stolen something?” [negatively keyed] or “From 1 to 10: How much does X like children?” [positively keyed]. A full list of selected items as well as details on the scoring can be found in Appendix Table 6. To measure the users’ prosocial, communal orientation, we z-standardized and averaged the users’ scores for agreeableness and narcissistic rivalry (reversed) from the second external survey (see Table 3). Based on the data of *n* = 1130 users with complete data for this analysis, as hypothesized, we found a significant positive correlation between the users’ communal orientation and the positivity of their responses, *r* = 0.14, *p* < 0.001.

At the level of targets, a common finding in person perception research is that first impressions are strongly guided by the target’s gender. Specifically, inferring a person’s gender quite automatically activates a variety of stereotypes regarding personality traits, skills, and preferences which heavily inform expectations about and impressions of that person (Bacev-Giles & Haji, 2017; Ellemers, 2018; Hack, 2014). To investigate whether this holds for the judgments collected in the Who Knows project, we used the male and female stereotypicality ratings of items (see above) to identify 20 stereotypically masculine items (i.e., items that were commonly expected to be endorsed by a typical male but not by a typical female) and 20 stereotypically feminine items (vice versa). The items are presented in Appendix Table 7. We then tested whether users’ judgments on these items were different depending on the gender of the target they referred to. When looking only at responses to stereotypically masculine items (13,793 responses by 3,104 users), as expected, the endorsement rate was 70% when the target gender was male and only 52% when the target gender was female, *d* = 0.39, *t* = 22.56, *p* < 0.001. Conversely, when looking only at responses to stereotypically feminine items (13,010 responses by 3,087 users), the endorsement rate was 63% when the target gender was female and only 39% when it was male, *d* = 0.50, *t* = 28.28, *p* < 0.001.

Finally, at the level of items, a central tenet of accuracy research has been that some personality items are more easily judgeable than others and that these differences manifest not only in differential accuracy rates (or item difficulties, for that matter) but also in differential levels of agreement between judges (i.e., consensus; Funder, 1995). In other words, when judges readily agree on how to judge targets on an item, these judgments should also be correct most of the time. In line with this, previous studies have found a strong positive correlation between consensus and accuracy (Funder & Dobroth, 1987; John & Robins, 1993). To replicate this, we computed the degree of invariance in users’ responses to each item as an index of consensus. The details of this computation differ for the different item formats and are explained in Appendix C. Crucially, the index is high when most respondents choose the same response option and low when they choose varying options. To index accuracy rates, we scored the percentage of correct responses to each item. As hypothesized, consensus and accuracy rates were substantially correlated across all item-types, *r*_binary_ = 0.41 (*n*_items_ = 221, *p* < 0.001), *r*_open-ended_ = 0.27 (*n*_items_ = 152, *p* < 0.001), *r*_scale_ = 0.60 (*n*_items_ = 137, *p* < 0.001), *r*_trivia_ = 0.46 (*n*_items_ = 297, *p* < 0.001).

In sum, these exemplary analyses demonstrate that the Who Knows data can be used to replicate existing findings concerning differences between judges (i.e., more prosocial users tend towards more evaluatively positive judgments), targets (i.e., female and male targets are judged differentially on items related to gender stereotypes) and items (i.e., items for which there is more agreement among judges are easier to judge).

## Opportunities for research and beyond

Beyond their ability to replicate existing, basic findings, the wealth of data collected within the Who Knows project may be highly informative for a variety of open research questions from methodological, basic, and applied subdisciplines of psychology and beyond. Other than in most existing research, the Who Knows data offer the valuable opportunity to tackle many of these research questions with multi-modal data, for instance by complementing the judgment data with cue-data about the targets (e.g., physical attractiveness) or behavioral data about the judge (e.g., time spent making the judgment). Table 5 provides a small collection of example research questions.

**Table 5:** Example Research Questions

Research area	Example research questions
Statistics	Cross-classified logistic regression models with planned missingness: Specification, estimation, coverage, and power
Psychometrics	Consistency, reliability, and stability of between-perceiver and between-target differences; scale construction based on dichotomous items tapping highly specific, and non-evaluative personality content
Personality	Antecedents and consequences of perceptive and expressive accuracy; factorial structure of personality nuances rarely covered in conventional inventories
Social Psychology	Brunswikian lens-model analyses on the validity and utilization of cues such as gender, age, profession, physical appearance, verbal utterances, gesture, posture, etc
Applied Psychology	Training effects in perceptive accuracy and moderators of training success

It is our explicit goal to share the Who Knows data with anybody from the scientific community who is interested in harnessing the data to investigate an empirical research question. Some of the data (i.e., data on targets and items) are openly accessible as part of the “Public data” component at https://osf.io/rdf7u/. In the same location, we also provide truncated versions of the user data (capped to 500 users), of the response data (capped to 100,000 responses), and of the logging data (capped to 200,000 logs) as well as a Codebook that documents the meaning of all variables in all data files. The non-truncated data on users, responses, and logs are stored in the repository’s non-public “App data” component and are automatically updated once a week. Access to this component can be requested from the first author via a request form available on the OSF.

Apart from harnessing the data that have already been collected, we would also like to encourage researchers to reach out to us with suggestions for how to make the Who Knows project even more scientifically useful in the future. Suggestions may include ideas for additional software features but also new items or scales to be included in the pool of quiz items or in one of the user surveys.

Beyond its utility for psychological research, the Who Knows project also advances the concept of *citizen science* (Bonney et al., 2014; Vohland et al., 2021) by having participants actively contribute to an ongoing, crowdsourced research effort whose purpose and rationale is communicated in an accessible and fully transparent way. In addition, participants can gain valuable insights into when and for whom their first impressions tend to be (in)accurate and potentially can even improve their judgmental skills. Finally, if the project can scale up its outreach, it promises a broader positive impact on society. Specifically, by offering users an opportunity to make low-level quasi-personal experiences with people from diverse sociodemographic backgrounds in a context that is appreciative and fun, the Who Knows project may contribute to more tolerance and depolarization in dealing with human diversity.

## Future extensions

We are constantly working to improve the Who Knows app and develop new features that may open up additional avenues for research to create additional scientific utility. Currently, we are rebuilding the app’s core architecture such that the app may obtain direct hardware access, most importantly to the devices’ cameras. This will allow users to become targets by uploading video content of themselves and will allow Who Knows to become an open and vivid platform with a much larger sample of targets than currently exists. This will not only enhance statistical power for research on between-target differences but also boost the gaming experience of high-frequency users, who will get to know more “fresh” targets as they use the app. Further, establishing camera access will also allow for an implementation of an eye tracking functionality that captures the users’ visual attention as they familiarize with the targets. This will contribute to an even richer body of behavioral data and will add value for psychological research on attention processes.

Another important feature on our to-do-list concerns an alternative game mode in which users do not only make a prediction of what they believe to be a target’s response on a given item but also rate how certain they are about this prediction. We believe that information about the users’ certainty may be valuable for many further research questions.

Finally, we plan to expand the Who Knows project such that the app becomes accessible by a more international audience. Specifically, once there exists an interface with which users can upload video content of themselves to become targets, there will be an option to upload either German or English content. Users can then choose from which language pool(s) they want their targets to be drawn. It is currently hard to predict when the above extensions will be fully implemented. Interested readers may visit https://whoknows.uni-muenster.de for updates.

## Data Availability

Data and materials of the Who Knows project are available from https://osf.io/rdf7u/.

## Appendix

See Table 6 and 7

**Table 6:** Selection of Items Used for Scoring Perceiver Positivity

ID	Item (free translation from German original)	Evaluativeness
205	Would X go return to a store if he/she found that he/she had received too much change?	0.90
22	Has X ever donated money to charity?	0.84
57	Does X sometimes park briefly in a disabled parking space? [reversed]	0.84
87	Has X ever cheated on someone? [reversed]	0.84
23	Has X ever secretly read a partner's text messages? [reversed]	0.82
9	Does X sometimes give money to beggars?	0.74
174	Has X broken curfew restrictions during the pandemic? [reversed]	0.74
102	Has X ever stolen something? [reversed]	0.70
171	Has X ever engaged in voluntary work?	0.70
200	Would X remind a friend that he/she still owes him/her one Euro? [reversed]	0.70
210	Has X ever secretly given a gift he/she received to someone else? [reversed]	0.70
10	Does X sometimes give money to street musicians?	0.66
107	Does X find it easy to apologize to others?	0.64
127	Is X often jealous of a friend? [reversed]	0.64
38	Does X hold grudges? [reversed]	0.62
98	Has X ever cheated on an exam? [reversed]	0.62
797	From 1 to 10: How much importance does X attach to outward appearances? [reversed]	0.90
715	From 1 to 10: How willingly would X influence others to achieve his/her goals? [reversed]	0.82
733	From 1 to 10: How happy is X about the misfortune of people he/she doesn't like? [reversed]	0.80
755	From 1 to 10: How likely is it that X secretly reads someone else's diary? [reversed]	0.78
734	From 1 to 10: How much does X enjoy the feeling of power? [reversed]	0.74
691	From 1 to 10: How sensitive is X to criticism? [reversed]	0.72
800	From 1 to 10: How optimistic is X?	0.70
814	From 1 to 10: How satisfied is X with him/herself and his/her current life?	0.70
711	From 1 to 10: How good is X at keeping a secret?	0.68
741	From 1 to 10: How much does X like children?	0.66
785	From 1 to 10: How important are the feelings of others to X?	0.64
771	From 1 to 10: How likely is it that X would start a bar fight? [reversed]	0.62
709	From 1 to 10: How good of a listener is X?	0.60
756	From 1 to 10: How likely is it that X will overturn the table if he/she has lost in a board game? [reversed]	0.60
808	From 1 to 10: How easy is it for X to accept his/her weaknesses?	0.60
717	From 1 to 10: How often does X use white lies? [reversed]	0.58

For upper half of the items, “yes” responses were coded as + 0.25 and “no” responses were coded as -0.25. For the lower half of the items, scale responses were transformed by the formula *f*(x) = (x – 5.5) / 10 such that they did not range from 1 to 10 but from -0.45 to + 0.45. After multiplying all responses to reverse-keyed items by -1, all of a user’s responses were averaged to obtain an index of positivity

**Table 7:** Selection of Items Identified as Tapping Gender Stereotypes

ID	Item (free translation from German original)	Stereotypicality rating		
		Male	Female	Δ
17	Does X have a favorite sports team?	0.88	0.06	0.81
67	Does X often take the lead in groups?	0.88	0.06	0.81
524	Does X know the main difference between diesel and gasoline engines?	0.88	0.13	0.75
192	Does X sometimes go to bed in his/her street clothes?	1	0.31	0.69
14	Does X own a pocket knife?	0.94	0.25	0.69
140	Does X sometimes leave dirty dishes at home until the next morning?	1	0.38	0.63
141	Did X skip school in the past?	0.94	0.31	0.63
116	Can X patch a bicycle tire?	0.88	0.25	0.63
150	Could X measure the oil level on a car?	1	0.44	0.56
33	Does X sometimes honk in the car to greet someone?	0.88	0.31	0.56
55	Does X often curse while driving?	0.88	0.31	0.56
88	Does X like to drive fast?	0.88	0.31	0.56
528	Does X know what the main function of a "web server" is?	0.69	0.13	0.56
472	Does X know how many rounds a professional boxing match usually lasts?	0.63	0.06	0.56
174	Did X illegally meet with several people during lockdown?	1	0.50	0.50
249	Does X know what the physical unit for measuring amperage is?	0.81	0.31	0.50
83	If X was given the chance to travel to the moon, would he/she do it?	0.75	0.25	0.50
99	Does X like to tell jokes?	0.75	0.25	0.50
167	Does X eat fast food at least once a week?	0.75	0.25	0.50
393	Does X know the name of the largest technology company in South Korea?	0.69	0.19	0.50
7	Does X like to go shopping for clothes with his/her partner?	0.06	1	-0.94
519	Does X know the difference between boots [German: "Stiefel"] and ankle boots [German: "Stiefeletten"]?	0.13	1	-0.88
25	Does X's favorite piece of clothing have a colorful color?	0.13	0.94	-0.81
506	Does X know what the fundamental difference between yoga and Pilates is?	0.00	0.81	-0.81
278	Does X know who wrote the "Inkheart Trilogy"?	0.19	0.94	-0.75
290	Does X know what a "butternut" is?	0.19	0.94	-0.75
143	Can X mend a hole in a pair of jeans?	0.13	0.88	-0.75
1	Does X get scared when he/she watches crime thrillers?	0.06	0.81	-0.75
106	Does X dwell on a mistake at work for a long time?	0.31	1	-0.69
94	Does X change the tone of his/her voice when he/she is on the phone?	0.25	0.94	-0.69
197	Does X like to dance at parties?	0.19	0.88	-0.69
481	Does X know what Bach flower remedies are used for?	0.19	0.88	-0.69
50	Does X spread toilet paper on public toilet seats before sitting down?	0.13	0.81	-0.69
144	Does X keep a pocket calendar?	0.13	0.81	-0.69
201	Does X like it when the hairdresser wants to chat while cutting hair?	0.13	0.81	-0.69
349	Does X know how Pamela Reif [German internet star] became famous?	0.13	0.81	-0.69
122	Does X like to dress up?	0.06	0.75	-0.69
321	Does X know which musical the word "supercalifragilisticepialigetisch" comes from?	0.06	0.75	-0.69
186	Does X like to make his/her own birthday cards?	0.00	0.69	-0.69
459	Does X know what the complementary color of yellow is?	0.00	0.69	-0.69

The upper half of items was classified as stereotypically masculine and the lower half of items was classified as stereotypically feminine

## Appendix C

For items with a categorical response format (types Binary, Open-ended, and Trivia), we computed consensus as the chi-square statistic that results from comparing the observed relative frequencies of responses with the relative frequencies that are expected under mere guessing (i.e., an even spread of frequencies across all response options). In case of a perfectly even spread, the chi-square statistics for an item is zero and the more the observed responses pile up on single response options, the larger the chi-square statistic. As such, the chi-square statistic is a straightforward index of consensus. For Scale-type items, we computed the inverse of the variance across all observed responses per item to index consensus.

It should be noted that neither are chi-square statistics comparable across items with a different number of response options nor are they comparable to our index of invariance for the Scale-type items. That is why we examined the relation between consensus and accuracy separately for each item type.

## Footnotes

1 Although the pool of potential items consists of 811 items, the number of items that have actually been responded to in a sufficient number of game rounds may be lower. This is because when a user starts a game, an algorithm first selects a random target and then a set of five items that are available for that target. However, the latter selection is constrained by several requirements that are meant to increase the heterogeneity of items within a game round to boost the gaming experience. For example, the first item is always of type Binary, two subsequent types cannot be of the same Type, etc. As a consequence of this non-random selection algorithm, some items are sampled quite rarely and others are sampled quite frequently. Nevertheless, the high overall amount of response data should render this imbalance largely unproblematic. For example, if one were to require that an item should have been responded to at least 100 times to be included in some substantive analysis, this requirement would be met by 730 of the 811 items as of fall 2023.

2 Because the distractors were manually generated, responses on the open-ended items may not exclusively be driven by the judges’ ability to infer characteristics of the target but also by their ability to take the perspective of those who generated the distractors (i.e., test-wiseness; Millman et al., 1965). Whether or not this constitutes a problem when analyzing the data depends on the substantive research question at hand. In case it does, responses to open-ended items should be excluded from the respective analyses.

## Code availability

R-code for the analyses reported as validity checks is available from https://osf.io/rdf7u/.

## Declarations

### Preregistration

These analyses reported as validity checks were not preregistered.
